# Long-Time Trend of Colorectal Cancer Mortality Attributable to High Processed Meat Intake in China and a Bayesian Projection from 2020 to 2030: A Model-Based Study

**DOI:** 10.3390/ijerph191710603

**Published:** 2022-08-25

**Authors:** Fangyao Chen, Shiyu Chen, Yaqi Luo, Aima Si, Yuhui Yang, Yemian Li, Weiwei Hu, Yuxiang Zhang

**Affiliations:** 1 Department of Epidemiology and Biostatistics, School of Public Health, Xi’an Jiaotong University Health Science Center, Xi’an 710061, China; 2 Department of Radiology, First Affiliated Hospital of Xi’an Jiaotong University, Xi’an 710061, China; 3 Department of Nursing, Xi’an Jiaotong University Health Science Center, Xi’an 710061, China

**Keywords:** colorectal cancer, processed meat, China, age-period-cohort analysis, Bayesian projection

## Abstract

Colorectal cancer is among the leading causes of cancer worldwide. Processed meat was known to be positively associated with a higher risk of gastrointestinal cancer. This study focused on the long-time trends of colorectal cancer mortality attributable to high processed meat intake in China from 1990 to 2019 and the projection for the next decade based on data obtained from the Global Burden of Disease 2019 study. We used an age-period-cohort model to fit the long-time trend. The joinpoint model was conducted to estimate the average and annual change of the attributable mortality. The Bayesian age-period-cohort model was used to project the crude attributable mortality from 2020 to 2030. An upward trend in colorectal cancer mortality attributable to high processed meat intake was observed for both sexes in China from 1990 to 2019, with an overall net drift of 4.009% for males and 2.491% for females per year. Projection analysis suggested that the burden of colorectal cancer incidence and mortality would still be high. Our findings suggested that colorectal cancer death attributable to high processed meat intake is still high in China, and elderly males were at higher risk. Gradually decreasing the intake of processed meat could be an effective way to reduce colorectal cancer mortality.

## 1. Introduction

Epidemiological studies have shown that the tumors in the digestive system account for about half of all malignant tumors worldwide [1]. Among those tumors, colorectal cancer (CRC) is one of the most frequent [1]. The incidence of CRC greatly varies in different countries and regions globally, and generally speaking, the CRC incidence is increasing in low- and middle-income countries, while it has generally remained stable or decreased in high-income countries [2]. Relevant studies have shown that there was about over 0.94 million CRC-caused cancer deaths in 2020 worldwide, ranking second among all malignant tumor deaths [1]. Globally, CRC has become a critical public health burden, and in Asia, its impact on the population’s health is increasing [3]. According to the latest released reports, the incidence of CRC continues to rise in China [4]. A relevant published study suggested that the prognosis of early-stage CRC is acceptable, with a 5-year survival rate of over 80%; however, the prognosis of advanced CRC is still far from satisfactory [5]. Therefore, to reduce the impact of CRC on the health of the population, preventing the occurrence of the disease has become very important.

Although the pathogenesis of CRC has not yet been fully understood, a study has shown that the initiation of CRC is closely associated with peoples’ living habits and dietary structure [6]. A healthy lifestyle can reduce CRC risk by 40%: regular exercise, avoiding smoking and drinking alcohol, increasing the intake of whole grains, and restricting the intake of red meat, especially processed meat (PM), are all important in preventing the onset of CRC [6]. A published study suggested that a diet high in fat, red meat, and PM is positively associated with increased CRC risk [7]. A recently published cohort study conducted in China also confirmed that the intake of meat, especially PM, is associated with the occurrence of CRC [8].

The negative impact of PM on human health has long been understood; however, the consumption of PM in many countries has shown no downward trend [9]. Considering the significant association between high PM intake and diseases, including CRC, many countries have officially proposed recommendations to restrict or reduce the intake of PM [10,11]. The International Agency for Research on Cancer (IARC) of the World Health Organization (WHO) has also officially classified PM as a carcinogen [12].

A published study has found that PM is associated with poor prognosis in CRC [13]. The processing methods and time may be the key element to their adverse health effects [14]. The use of nitrite, the smoking process, etc., may all play important roles in carcinogenesis [15]. Additionally, when producing PM, the proportion of fat can range from 10% to 35%, and if it is bacon, the proportion of fat may be higher. Previous studies have shown that increased intake of high-fat foods can promote bile secretion and increase the concentration of bile acids in the intestine, which in turn activates the Wnt signaling pathway and increases the risk of colorectal cancer [16]. Studies also have shown that PM can increase the chance of local inflammation in the intestine; activate the P13K-AKT-MTOR pathway, which can induce or stimulates cell overgrowth, proliferation, and migration; and can also inhibit the body’s anti-tumor immune response and achieve immune escape, thereby promoting the occurrence and progression of CRC [7,17,18,19].

Though it is well-acknowledged that the high intake of PM is associated with increased CRC risk, within the literature review, we found few published studies focused on CRC mortality attributable to high PM intake. Therefore, in this study, we aimed at the long-time trend of CRC mortality attributed to high PM intake in China and the comparison of sex differences. Our analysis was conducted based on the data obtained from the Global Burden of Disease (GBD) 2019 study. Under the age-period-cohort (APC) frame, we comprehensively analyzed the effects of age, year period, and birth cohort, thereby exploring the long-time trends and corresponding sex difference in CRC mortality attributed to high PM intake in China. A Bayesian age-period-cohort (BAPC) model was then conducted to predict the changes in CRC incidence and mortality in China by 2030.

## 2. Materials and Methods

### 2.1. Research Data

The study data were all obtained from the GBD 2019 study using the Global Health Data Exchange Tool (http://ghdx.healthdata.org/, accessed on 15 March 2022). The GBD 2019 study is an international collaboration led by the Institute for Health Metrics and Evaluation [20]. The program provides comprehensive, guidelines-based, summarized survey statistics on traits and degrees of health outcomes and corresponding risk factors [20]. The GBD 2019 study contained survey statistics from 204 main countries and territories and the first administrative level areas collected from 22 countries from 1990 to 2019 [20].

### 2.2. Definitions of Measurements

In the GBD 2019 study, a diet high in PM is defined as an average daily consumption (in grams) of more than 2 grams (0–4 grams) of PM, of which, PM refers to meat preserved by smoking, curing, salting, or addition of chemical preservatives [20,21].

According to the design of the GBD 2019 study, the survey statistics of CRC mortality in China were collected from the Disease Surveillance Points, the Maternal and Child Surveillance System, and the Chinese Center for Disease Control and Prevention Cause of Death Reporting System in China [21,22]. 

The CRC mortality was calculated according to the International Statistical Classification of Diseases and Related Health Problems, Ninth Revision (ICD-9) or Tenth Revision (ICD-10) [23,24]. The high-PM-intake-attributed CRC mortality rates were defined using the population-attributable fractions (PAF) considering sexes, year periods, and age groups. The general trends of crude mortality rates (CMR) and age-standardized mortality rates (ASMR) per 100,000 populations were also analyzed in this study.

### 2.3. Statistical Analysis

Graphs were generated with R programming language (version 4.0.3, The R Foundation, Vienna, Austria) and RStudio (Version 1.1.463, RStudio Inc., Boston, MA, USA) software. All statistical tests were 2-tailed, and the level of significance was set at 0.05. 

#### 2.3.1. Age-Period-Cohort Model

To estimate the effect of age, year period, and birth cohort, we performed the age-period-cohort (APC) model. To convert the data to fulfill the requirement of the model, the age-specific CRC mortality rates attributable to high PM intake were organized into consecutive 5-year periods from 1990 to 2015 and successive 5-year age groups from the age of 25 to 29 years old to 80 to 84. 

The APC model can be expressed as follows [25]:

$$R_{ijk}=\mu +\alpha_{i}+\beta_{j}+\gamma_{k}$$

In which μ is the constant, and $R_{ijk}$ represents the attributable mortality rate in the *i*th age group, *j*th time period, and *k*th birth cohort. $\alpha_{i}$, $\beta_{j}$, and $\gamma_{k}$ are the effects of age, period, and cohort, respectively. In the APC analysis, the relative risk (RR) is defined as the exponential value of the estimations of $\alpha_{i}$, $\beta_{j}$, and $\gamma_{k}$. 

We also used the APC model to estimate the net drifts, the local drifts, the longitudinal curves, and their corresponding 95%CI. The net drifts and longitudinal curves were estimated and adjusted for the effects of the time period and birth cohort in the APC model. 

We then estimated the net and local drift, which stands for the overall log-linear trend by year period and birth cohort. The adjusted relative risk of year period and birth cohort were also estimated. The estimation of the APC model was conducted through the APC Web Tool (https://analysistools.nci.nih.gov/apc/, accessed on 17 March 2022) [26].

#### 2.3.2. Joinpoint Analysis

The long-time trends of CRC mortality attributable to high PM intake were explored through the joinpoint regression, which is also called the multi-phase regression. We divide its long-time trend into several segments and estimated the average percent change (AVPC) and average annual percent change (AAPC) with a corresponding 95% confidence interval (CI). The joinpoint regression model can be defined as:

$$E(y_{i})=\zeta_{0}+\zeta_{1}T+\delta_{1}{\left(x_{i}-\tau_{1}\right)}^{+}+\ldots +\delta_{k}{\left(x_{i}-\tau_{n}\right)}^{+}$$

In which $\zeta_{0}$ is the constant. $y_{i}$ represents to attributable mortality rate, and T is the observation year. $\zeta_{1}$ represents the coefficient of *T*, and $\tau_{n}$ is the unknown turning time point (joint point) that needs to be identified.

The joinpoint regression was conducted with the Joinpoint Regression Program (version 4.9.0.0, Statistical Research and Application Branch, NCI, Bethesda, MD, USA). 

#### 2.3.3. Bayesian Age-Period-Cohort Analysis

The prediction analysis was conducted with the Bayesian age-period-cohort (BAPC) model [27]. Age-specific CRC attributable to high PM intake of lung cancer in China from 2020 to 2030 was predicted using the BAPC model with integrated nested Laplace approximations [27]. The ordinary APC model was defined as in Section 2.3.1, and the effects of age in a BAPC model can be estimated as:

$$f\left(\alpha |l_{\alpha }\right)\propto l_{\alpha }{}^{\frac{I-2}{2}}\exp\left[-\frac{l_{\alpha }}{2}\sum_{i=3}^{I}{\left(\alpha_{i}-2\alpha_{i-1}+\alpha_{i-2}\right)}^{2}\right]$$

In which$\;l_{\alpha }$ is the variance parameter. To avoid the issue of over dispersion, an independent random effect $z_{ij}\sim N\left(0,\kappa_{z}^{-1}\right)$ is added to the basic APC model for a particular *i*th age group with time period *t* for the following years as follows:

$$R_{ijk}=\mu +\alpha_{i}+\beta_{j+t}+\gamma_{k+t}+z_{ij+t}$$

And the effects of the period in period *j* + 1 are assumed to follow the distribution as follows:

$$\beta_{j+1}|\beta_{1},\ldots ,\beta_{j},\;\kappa_{\beta }\sim N\left(2\beta_{j}-\beta_{j-1},\kappa_{\beta }^{-1}\right)$$

The data from 1990 to 2019 were used to train the model. The model was fitted using the R package “BAPC” (version 0.0.34) [27].

## 3. Results

### 3.1. General Trend of the Attributed CRC Mortality

**Figure 1:**
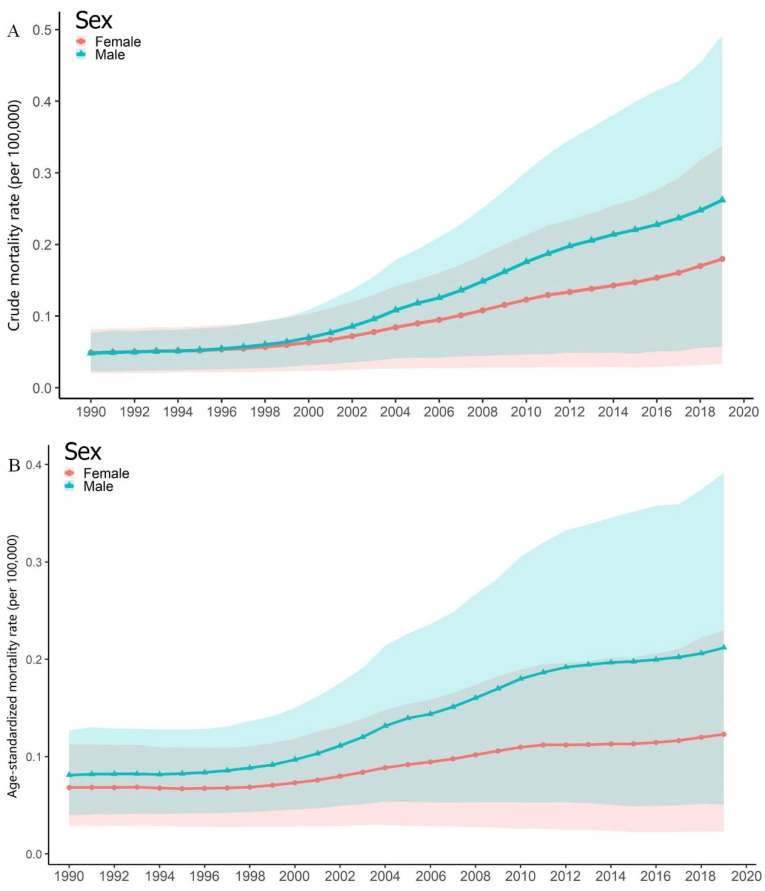
Crude mortality rates (CRMs) and age-standardized mortality rates (ASMRs) of CRC (per 100,000) from 1990 to 2019 and corresponding 95% confidence intervals (presented with shadows) attributable to high PM intake for both sexes in China. (**A**) CMRs for both sexes; (**B**) ASMRs for both sexes.

The long-term trend of CRC ASMR attributable to high PM intake in China considering sex differences is presented in Figure 1. As shown in Figure 1A, the attributable CMR for both sexes in China shows an increasing trend. To be specific, the CMR for females increased from 0.049 (95%CI: 0.020~0.082) per 100,000 to 0.180 (95%CI: 0.023~0.337) from 1990 to 2009 and from 0.048 (95%CI: 0.023~0.077) to 0.262 (95%CI: 0.057~0.492) per 100,000 for males, respectively. However, considering the overlaps of the confidence intervals and the CMRs in each year for both sexes, the increase may not be statistically significant.

For the attributable ASMRs in China, as shown in Figure 1B, an increasing trend from 1990 to 2019 for both sexes was observed, too. To be specific, the ASMR for females increased from 0.068 (95%CI: 0.029~0.112) to 0.113 (95%CI: 0.023~0.229) per 100,000 and from 0.081 (95%CI: 0.040~0.127) to 0.212 (95%CI: 0.051~0.391) per 100,000 for males. However, considering the overlaps of the confidence intervals and the CMRs in each year for both sexes, the increase may not be statistically significant. 

### 3.2. Local and Net Drifts

**Figure 2:**
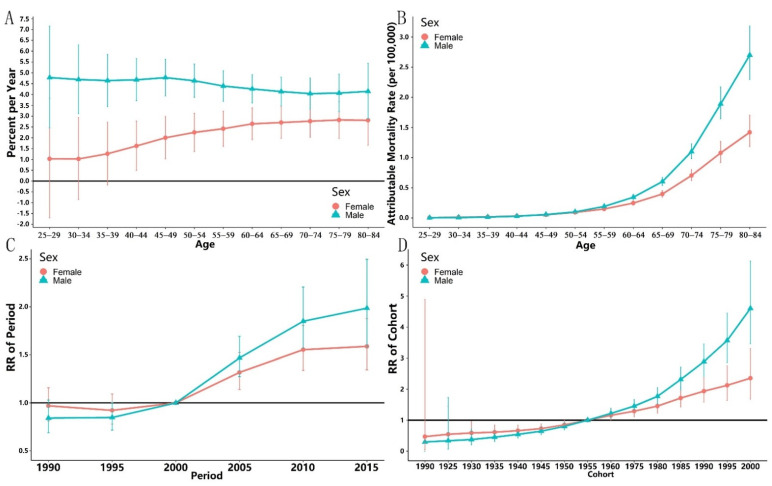
Local drifts, longitudinal age curves, estimated relative risk (RR) for period and cohort, and corresponding 95%CI for both sexes in China. (**A**) Local drifts for both sexes; (**B**) longitudinal age curve; (**C**) period RR; (**D**) cohort RR.

Figure 2A presents the local drifts of the CRC mortality attributable to high PM intake for both sexes in China, respectively. Generally speaking, the local drifts for males is about two times higher than that of females. As shown in Figure 2A, the local drifts for males and females in China show a relatively stable trend (above 0) across age groups, while slight decreases were also observed for the age group 60–64 and above for both sexes. The local drifts for age groups 65–69 and 70–74 were not significantly different from 0 (*p* > 0.05) for both sexes.

The overall net drift is 4.009% (95%CI: 2.708~5.489%; *p* < 0.001) for Chinese males and 2.491% (95%CI: 1.704~3.285%; *p* < 0.001) for females. 

### 3.3. Age-Period-Cohort Analysis

The longitudinal effect of age is presented in Figure 2B. The CRC mortality rates (per 100,000) attributable to high PM intake show an increasing trend along with age groups for both sexes. Overall, the increasing trend for males in China is more rapid than that of females. For males, the mortality rates increased from 0.056% (95%CI: 0.046~0.069%) to 5.427% (95%CI: 1.671~17.625%) for the age groups of 25–29 to 70–74 (*p* < 0.001) though the increasing trend slowed down at the age group of 70–74. For females, the mortality rates increased from 0.0617% (95%CI: 0.049~0.077%) to 2.678% (95%CI: 1.511~2.637%) for the age groups of 25–29 to 70–74 (*p* < 0.001). 

The estimated period RRs by sex are shown in Figure 2C. The period RR for both sexes in China shows an approximately monotonic increasing trend. The reference year was the year 2000. The estimated period RRs before the year 2000 were not statistically significant (*p* > 0.05) for both sexes, while thereafter, the estimated period RRs were all statistically higher than 1 (*p* < 0.05) and show an increasing trend.

The estimated cohort RRs by sex are shown in Figure 2D. The cohort RRs for Chinese males and females both show a significant upward trend across birth cohorts, while that for Chinese females is lower than that of males. The estimated cohort RRs were not statistically different across sexes before the year 1970, while thereafter, the increasing speed of cohort RRs for males was much higher than that of females.

### 3.4. Joinpoint Analysis

The result of the joinpoint analysis was presented in Table 1. There are six trends for the ASMRs of females in China, with an average increase (AAPC) of 2.0% (95%CI: 1.9~2.2%, *p* < 0.001) per year from trend 1 to trend 6 (1990~2019). The peak of growth appears in trend 3 from 2000 to 2004, at 5.2% (95%CI: 4.5~5.8%, *p* < 0.001) per year. For the CMRs of females in China, an upward trend was observed (*p* < 0.001). The peak of growth appears in trend 3 from 2001 to 2004, at 8.1% (95%CI: 6.9~9.2%, *p* < 0.001) per year. 

For the ASMRs of males in China, the overall trend is upward; the AAPC is 3.3% (95%CI: 3.0~3.7%) per year. The peak appears in trend 3 from 2000 to 2004, at 8.1% (95%CI: 6.6~9.7%, *p* < 0.001). For the CMRs of males in China, the general trend is upward (*p* < 0.001). The peak of growth appears in trend 3 from 2000 to 2004, at 11.9% (95%CI: 10.6~13.3%, *p* < 0.001) per year, as presented in Table 1.

### 3.5. Bayesian Prediction

**Figure 3:**
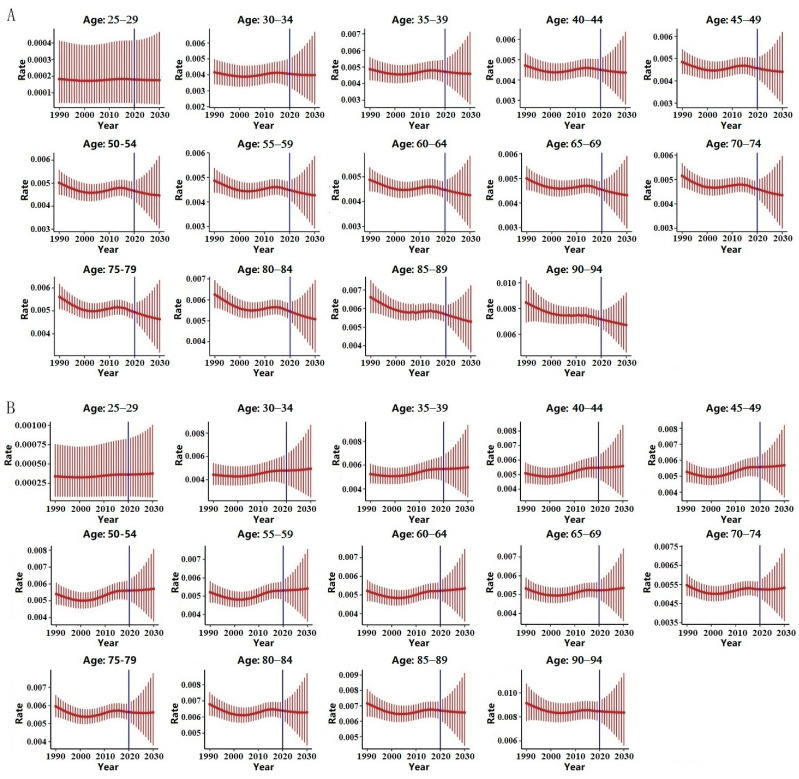
Projected age-specific CRC crude mortality rates (CMRs) based on Bayesian age-period-cohort analysis for both sexes in China from 2020 to 2030. (**A**) Projected CMRs for males; (**B**) projected CMRs for females.

We then conducted a Bayesian age-period-cohort analysis to project future age-specific crude CRC mortality rates attributable to high PM intake for both sexes in China, and the results are presented in Figure 3 and Table 2 and Table 3. Figure 3A (for males) and Figure 3B (for females) illustrate the projected age-specific attributable CMRs from 2020 to 2030. 

Our results indicated that for males, the age-specific attributable CMRs in China would continue to decrease from 2020 to 2030s though the trend was not statistically significant. The deceasing trend were consistent across different age groups. As shown in Figure 3A and Table 2, the attributable CMRs decreased from 2019 to 2030.

For females, the age-specific attributable CMRs would slightly increase for the age groups before 75 to 79 though the changes were not statistically significant. For females in age groups after (and including) 75 to 79, the projected attributable CMRs decreased from 2019 to 2030; however, the trend was not statistically significant, as shown in Figure 3B and Table 3.

## 4. Discussion

In this study, we estimated age, period, and cohort effects in CRC mortality attributable to high PM intake between 1990 and 2019 using classic age-period-cohort analyses; quantified the annual and overall average changes from 1990 to 2019 using the joinpoint regression; and projected the attributable CRC CMR from 2020 to 2030 through a Bayesian age-period-cohort analysis with integrated nested Laplace approximations by sexes in China. 

We found increasing trends of the age, cohort, and period effects for CRC mortality attributable to high PM intake in China for both sexes during the past decades. The net and local drifts indicated that the CRC risk attributable to high PM intake for both sexes in China is still increasing. The projected future age-specific CMR by sex also indicated that the CRC mortality attributable to high PM intake will still be high in the next decade. Our results suggested that the CRC mortality attributable to high PM intake is still not under control. 

The attributable CMRs and ASMRs both showed an upward trend for both sexes in China. The APCs and AAPCs estimated with the joinpoint analysis suggested the same trend. A significant sex difference was also observed. A published study has suggested that CRC mortality in males is higher than in females [28]. This is partly because males were more likely to be associated with CRC risk factors such as alcohol, smoking, and red meat or PM [29]. More exposure to those risk factors may increase the mortality risk. 

It is well-acknowledged that age is positively associated with the initiation and progression of multiple kinds of cancer. In this study, similar trends of the age effects on CRC mortality attributable to high PM intake were obtained for Chinese males and females during the last three decades, while the age effect of males is higher than that of females. This age effect could be partly due to the worsening outcomes and prognosis of CRC along with the increase of age [30] and partly because elder adults may have weaker digestive functions than the young [31]. A published study suggested that CRC incidence increases along with age; the CRC incidence is at a low level in young adults, while it raises rapidly with age and reaches a peak after the age of 80 [32].

The RR of age increased from the age group 25–29 to the age group of 80–84 for both sexes and shows no slowing trend, indicating that aging also accelerates the increasing trend of attributable mortality. Moreover, this result also suggests that elder adults may need closer monitoring to discontinue the increasing trend of attributable mortality, especially for males in China. 

Our analysis also suggested that the period RRs have increased in Chinese adults for the past several years. This could be explained by the increasing consumption of PM in China [33]. In this study, we found that the CRC mortality attributable to high PM intake in China for both sexes had increased significantly over the past three decades. Although significant improvements in CRC diagnosis, treatment, and patients’ management have achieved, the attributable CRC mortality did not show a decreasing trend. 

In this study, we also found that the effect of the birth cohort also showed an increasing trend and suggested that lately, birth cohort suffers a higher CRC risk attributable to high PM intake. The effect of the birth cohort usually indicates exposure to risk factors in early life. Compared with the older generations, young people consume more PM and PM products [34,35,36,37]. These results suggest that it is urgent to conduct more effective health education for residents in China and guide them to establish healthy eating habits, especially for the young generations.

In Bayesian age-period-cohort analysis, the projected age-specific attributable CMRs from 2020 to 2030 are not consistent across sexes and age groups. For both sexes in age groups after 70 to 74, the attributable CMRs showed a downward trend (though without statistical significance). In recent years, with the rapid development and clinical application of new therapies such as immunotherapy and neoadjuvant chemotherapy, the basic CMRs of CRC have been decreasing [38,39], and the attributable mortality is proportional to its crude mortality [40], thus also showing a downward trend. 

As suggested by the “China guideline for the screening, early detection and early treatment of colorectal cancer”, CRC screening for the general population starts at age 40 [28]. The implementation of nationwide CRC screening has greatly promoted the early detection, diagnosis, and treatment of colorectal cancer and reduced the death caused by CRC. The projection analysis may suggest that the current screening policy, including the released dietary guidelines, may be needed to continue to ensure the control of attributable mortality. 

In the analysis, we also found that for age groups before (and including) 70–74, the projected CMRs of the females showed a slight upward trend (but no statistical significance). Published study has confirmed that female hormones, estrogen replacement therapy, and oral contraceptives are associated with decreased risk of CRC initiation [41]. However, though with a lower incidence, females are far less likely to be screened for CRC than males, especially for the young and middle-aged [42]. Low participation in CRC screening may result in CRC patients being diagnosed at a late stage, potentially leading to a poor prognosis. Furthermore, the incidence of early-onset CRC has continued to increase worldwide, and the youngest CRC patients were diagnosed at a late stage, which leads to an unfavorable prognosis [43]. 

Increased incidence of CRC in young adults and inadequate screening for CRC in females may also contribute to increased CRC mortality in young and middle-aged women, as projected. Moreover, compared with men, early symptoms of CRC in women are more likely to be confused with diseases of the female reproductive system, leading to misdiagnosis [44]. The increasing trend of the projected age-specific attributable mortality for Chinese females suggests that more attention should be paid to enhancing early screening of potential female CRC patients.

As shown in Figure 1, the high-PM-intake-attributable CRC mortality has been increasing over the past few decades though no statistical significance was obtained. A previously published study suggested that the mortality of chronic diseases is relatively stable, especially for malignant tumors, whose occurrence and development require a long-term process [45]. In a relatively fixed population, the mortality changes are not likely to fluctuate too much within a certain period of time [45]. The BAPC model used a MCMC approach to estimate parameters and therefore could smooth the effects of age, time period, and birth cohort and avoid the existence of excessive fluctuation in projection [46]. 

Although the Chinese Dietary Guidelines published by the Chines Nutrition Society have recommended controlling and reducing the intake of PM [11], the consumption of PM and PM products in China has greatly increased during the past several decades and was predicted to remain a stable, non-decreasing trend in the next 10 years [47]. It was reported recently that Asia had the highest CRC incidence and mortality for all sexes and ages (per 100,000 populations) in the world [48]. A published study also revealed that in 2020, there were about 4.57 million new cancer cases in China, and the incidence of CRC jumped to second place, following only lung cancer [49]. In addition, in 2020, about 3 million people will die from cancer in China, and CRC is the fifth most common cause of cancer death [49]. These all suggest that we need to strengthen the healthy guidance on the daily eating habits of Chinese residents and guide the public to rationally control the consumption of PM. Furthermore, though males were at higher risk than females, attention should also be paid for the females (for example, by encouraging female adults to participate in early screening for CRC) to control the possible upward trend of the attributable mortality. 

Our study has some limitations. Firstly, this study was conducted based on the summarized data obtained from the GDB 2019 database but not on individual-level data. This limitation prevents us to analyze the different impacts of other social-economical characteristics (except sexes) on mortality. At the same time, the comparability of the attributable mortality in different provinces in China may be of practical meaning; however, the province-level data were not available, and future study may focus on this issue. In addition, the Bayesian and classic age-period-cohort analysis both require a fixed age and time period, and therefore, people aged over 95 years were removed. In the age-specific projection, the confidence intervals are large, indicating large errors. To be specific, the estimated errors increased over time. Within the literature review, we found this may be because the data were split into different age groups, resulting in a small base for estimated rates on each group, and this may enlarge the standardized errors of rates and lead to large confidence intervals [27,50,51,52]. Despite the uncertainty in projection analysis, the long-term projection could provide useful information in the management of future medical source allocation and policy making.

## 5. Conclusions

In this study, we found that the overall CRC mortality burden attributable to high PM intake in China is high currently and is projected to still be high in the next decade. A significant sex difference was also observed: males were at higher risk compared to females, especially for those aged residents in China. Our findings suggested that gradually controlling and decreasing the intake of PM and PM products may provide a positive effect in reducing CRC mortality.

## Figures and Tables

**Table 1:** Average and annual percent changes (AVPCs and AAPCs) of CRC mortality attributable to high PM intake.

Age-Standardized Mortality Rates (ASMRs)					Crude Mortality Rates (CMRs)		
		Time Period	AVPC (95%CI)	*t* (*p*)	Time Period	AVPC (95%CI)	*t* (*p*)
Female	Trend 1	1990~1997	−0.2 (−0.4,0)	−2.7 (0.020)	1990~1997	1.5 (1.3,1.7)	15.0 (<0.001)
	Trend 2	1997~2000	2.5 (1.1,3.9)	3.9 (0.002)	1997~2001	5.4 (4.7,6.1)	17.2 (<0.001)
	Trend 3	2000~2004	5.2 (4.5,5.8)	17.4 (<0.001)	2001~2004	8.1 (6.9,9.2)	15.4 (<0.001)
	Trend 4	2004~2011	3.5 (3.3,3.7)	41.5 (<0.001)	2004~2011	6.4 (6.2,6.5)	97.0 (<0.001)
	Trend 5	2011~2016	0.2 (−0.1,0.5)	1.5 (0.146)	2011~2016	3.3 (3.1,3.5)	34.0 (<0.001)
	Trend 6	2016~2019	2.6 (2.1,3.2)	11.0 (<0.001)	2016~2019	5.6 (5.3,5.9)	40.8 (<0.001)
	AAPC	-	2.0 (1.9,2.2)	22.7 (<0.001)	-	4.6 (4.4,4.8)	59.2 (<0.001)
Male	Trend 1	1990~1996	0.3 (−0.4,1.0)	1.0 (0.342)	1990~1996	1.8 (1.1,2.5)	5.7 (<0.001)
	Trend 2	1996~2000	3.6 (1.8,5.5)	4.3 (0.001)	1996~2000	6.3 (4.6,8.1)	8.2 (<0.001)
	Trend 3	2000~2004	8.1 (6.6,9.7)	11.3 (<0.001)	2000~2004	11.9 (10.6,13.3)	20.2 (<0.001)
	Trend 4	2004~2011	5.4 (5,5.8)	28.7 (<0.001)	2004~2011	8.4 (8.2,8.7)	66.8 (<0.001)
	Trend 5	2011~2019	1.4 (1.1,1.6)	12.7 (<0.001)	2011~2017	3.8 (3.5,4)	30.9 (<0.001)
	Trend 6	-	-	-	2017~2019	5.0 (3.9,6.1)	10.3 (<0.001)
	AAPC	-	3.3 (3,3.7)	19.2 (<0.001)	-	6.0 (5.7,6.3)	38.2 (<0.001)

**Table 2:** Projected mortality rate and corresponding 95%CI for males in selected years.

Age Group		Rate (95%CI)	
	2019	2020	2030
25–29	0.00018 (0.00004,0.00041)	0.00018 (0.00004,0.00041)	0.00018 (0.00003,0.00046)
30–34	0.00408 (0.00331,0.00495)	0.00406 (0.00323,0.00502)	0.00399 (0.00217,0.00667)
35–39	0.00474 (0.00411,0.00541)	0.00472 (0.00402,0.00548)	0.00460 (0.00277,0.00710)
40–44	0.00452 (0.00404,0.00503)	0.00450 (0.00397,0.00507)	0.00437 (0.00282,0.00636)
45–49	0.00458 (0.00418,0.00501)	0.00457 (0.00411,0.00505)	0.00441 (0.00296,0.00618)
50–54	0.00469 (0.00433,0.00507)	0.00466 (0.00424,0.00511)	0.00447 (0.00305,0.00617)
55–59	0.00450 (0.00418,0.00484)	0.00448 (0.00409,0.00488)	0.00427 (0.00293,0.00586)
60–64	0.00450 (0.00419,0.00482)	0.00447 (0.00410,0.00487)	0.00426 (0.00293,0.00583)
65–69	0.00457 (0.00427,0.00488)	0.00454 (0.00417,0.00492)	0.00431 (0.00297,0.00590)
70–74	0.00464 (0.00435,0.00494)	0.00461 (0.00425,0.00500)	0.00436 (0.00301,0.00595)
75–79	0.00498 (0.00466,0.00530)	0.00494 (0.00455,0.00535)	0.00464 (0.00320,0.00633)
80–84	0.00549 (0.00514,0.00586)	0.00545 (0.00501,0.00590)	0.00508 (0.00350,0.00694)
85–89	0.00573 (0.00532,0.00616)	0.00568 (0.00520,0.00619)	0.00530 (0.00365,0.00724)
90–94	0.00721 (0.00661,0.00784)	0.00717 (0.00649,0.00788)	0.00672 (0.00462,0.00922)

**Table 3:** Projected mortality rate and corresponding 95%CI for females in selected years.

Age Group		Rate (95%CI)	
	2019	2020	2030
25–29	0.00036 (0.00009,0.00082)	0.00036 (0.00008,0.00083)	0.00038 (0.00007,0.00100)
30–34	0.00478 (0.00368,0.00606)	0.00478 (0.00360,0.00618)	0.00495 (0.00250,0.00868)
35–39	0.00566 (0.00473,0.00670)	0.00567 (0.00465,0.00682)	0.00581 (0.00330,0.00933)
40–44	0.00547 (0.00475,0.00625)	0.00547 (0.00469,0.00633)	0.00558 (0.00344,0.00840)
45–49	0.00557 (0.00497,0.00621)	0.00557 (0.00491,0.00630)	0.00568 (0.00368,0.00818)
50–54	0.00560 (0.00506,0.00617)	0.00560 (0.00500,0.00625)	0.00571 (0.00378,0.00805)
55–59	0.00530 (0.00485,0.00577)	0.00530 (0.00478,0.00586)	0.00541 (0.00362,0.00756)
60–64	0.00521 (0.00479,0.00566)	0.00523 (0.00473,0.00575)	0.00535 (0.00360,0.00744)
65–69	0.00522 (0.00482,0.00564)	0.00523 (0.00475,0.00573)	0.00535 (0.00361,0.00743)
70–74	0.00525 (0.00486,0.00565)	0.00525 (0.00478,0.00574)	0.00532 (0.00359,0.00737)
75–79	0.00565 (0.00524,0.00607)	0.00564 (0.00514,0.00616)	0.00561 (0.00380,0.00778)
80–84	0.00641 (0.00594,0.00690)	0.00639 (0.00582,0.00699)	0.00628 (0.00425,0.00871)
85–89	0.00671 (0.00619,0.00727)	0.00669 (0.00607,0.00735)	0.00657 (0.00443,0.00910)
90–94	0.00847 (0.00772,0.00926)	0.00847 (0.00762,0.00937)	0.00836 (0.00563,0.01162)

## Data Availability

Data used in this study were all obtained from the GBD 2019 study using the Global Health Data Exchange Tool (http://ghdx.healthdata.org/, accessed on 15 March 2022).
